# Platelet Activation in Heparin-Induced Thrombocytopenia is Followed by Platelet Death via Complex Apoptotic and Non-Apoptotic Pathways

**DOI:** 10.3390/ijms21072556

**Published:** 2020-04-07

**Authors:** Elmira R. Mordakhanova, Tatiana A. Nevzorova, Gulnaz E. Synbulatova, Lubica Rauova, John W. Weisel, Rustem I. Litvinov

**Affiliations:** 1 Institute of Fundamental Medicine and Biology, Kazan Federal University, Kazan, Tatarstan 420008, Russia; elmira.m.m@yandex.ru (E.R.M.); tnevzorova@gmail.com (T.A.N.); rustempa@gmail.com (G.E.S.); 2 The Children’s Hospital of Philadelphia, Philadelphia, PA 19104, USA; lubica@email.chop.edu; 3 Departments of Pediatrics, University of Pennsylvania School of Medicine, Philadelphia, PA 19104, USA; weisel@pennmedicine.upenn.edu; 4 Departments of Cell and Developmental Biology, University of Pennsylvania School of Medicine, Philadelphia, PA 19104, USA

**Keywords:** platelets, heparin-induced thrombocytopenia, platelet death, apoptosis, caspase, calpain

## Abstract

Heparin-induced thrombocytopenia (HIT) is an adverse drug reaction characterized by thrombocytopenia and a high risk for venous or arterial thrombosis. HIT is caused by antibodies that recognize complexes of platelet factor 4 and heparin. The pathogenic mechanisms of this condition are not fully understood. In this study, we used flow cytometry, fluorimetry, and Western blot analysis to study the direct effects of pathogenic immune complexes containing platelet factor 4 on human platelets isolated by gel-filtration. HIT-like pathogenic immune complexes initially caused pronounced activation of platelets detected by an increased expression of phosphatidylserine and P-selectin. This activation was mediated either directly through the FcγRIIA receptors or indirectly via protease-activated receptor 1 (PAR1) receptors due to thrombin generated on or near the surface of activated platelets. The immune activation was later followed by the biochemical signs of cell death, such as mitochondrial membrane depolarization, up-regulation of Bax, down-regulation of Bcl-X_L_, and moderate activation of procaspase 3 and increased calpain activity. The results show that platelet activation under the action of HIT-like immune complexes is accompanied by their death through complex apoptotic and calpain-dependent non-apoptotic pathways that may underlie the low platelet count in HIT.

## 1. Introduction

Heparin-induced thrombocytopenia (HIT) is an immune-mediated adverse reaction to heparin, a broadly used anticoagulant [1]. Heparin exposure leads to the formation of IgG antibodies (Abs) that recognize multimolecular complexes of platelet factor 4 (PF4) and heparin that form in the blood and on the surface of platelets and other cells [2]. These immune complexes bind to the FcγRIIA receptors of platelets, resulting in platelet activation associated with the exposure of procoagulant phosphatidylserine (PS) on the platelet membrane and on platelet-derived microparticles [3] as well as the expression of P-selectin [4]. Alternatively, platelets are transactivated by thrombin which is generated by monocytes [5]. Irrespective of the underlying mechanism, platelet activation is central to the pathogenesis of HIT. The end result is the development of venous and arterial thromboses associated with a persistent low platelet count.

Despite a lot of information being available on triggering factors and pathogenic mechanisms involved in HIT, the causes of thrombocytopenia in HIT are not well delineated. One possibility is that a low platelet count is triggered by the clearance of Ab-coated platelets in the spleen and potentially the liver. The second possibility is that platelets are consumed within thrombi; however, it is unclear if sufficient platelets are incorporated into thrombi to cause thrombocytopenia. The third potential explanation involves platelet disintegration via microvesiculation [6,7], but whether this mechanism is responsible for the low platelet count is unclear. Our recent studies suggest that platelets activated by thrombin undergo fatal dysfunction and cytoplasmic fragmentation clearly distinct from what happens when platelets are activated by adenosine diphosphate (ADP) or collagen [8,9]. Based on these and other findings, we hypothesized that platelets activated in HIT through FcγRIIA undergo activation followed by death, leading to thrombocytopenia with the removal of dead platelets perhaps by neutrophils and macrophages. 

In this work, we studied the direct effects of PF4-containing HIT-pathogenic immune complexes on isolated human platelets. As a tool, we used two isotype-matched murine anti-human PF4/heparin monoclonal Abs that mimic their human counterparts in vitro [10], and in vivo the pathogenic monoclonal anti-PF4/heparin antibody (KKO) causes HIT in an animal model, while the non-pathogenic monoclonal anti-PF4/heparin antibody (RTO) does not [11]. Importantly, ELISA-positive plasma samples from patients suspected of having HIT contain Abs that show heparin-induced binding to PF4, like KKO, and compete with KKO for binding to heparin/PF4 and activate platelets in a heparin- and FcRγIIA-dependent manner. However, samples that are ELISA-positive but do not activate platelets behave like RTO, show less inhibition in KKO binding to PF4, and do not activate platelets [12]. KKO and RTO do not compete for binding to PF4. KKO, unlike RTO, causes PF4 to oligomerize in solution, forming ultra-large complexes that cluster on cell surfaces, which probably activate the cells and predispose to Ab-induced thrombosis [13]. This suggests that KKO is an appropriate model Ab for studies of HIT pathology, and comparisons between KKO and RTO might help to define the difference between the pathogenic and non-pathogenic human anti-PF4 Abs that underlie their dissimilar clinical impact.

Using flow cytometry and Western blot analysis, we showed that, after activation, platelets undergo a complex death pathway that combines molecular signs of apoptosis and activation of calpain.

## 2. Results and Discussion

### 2.1. Subpopulations of Live Activated and Dying Platelets

To reproduce platelet perturbations in HIT, isolated human platelets were treated in vitro for 15 and 60 min with a combination of PF4 and pathogenic (KKO) or non-pathogenic (RTO) monoclonal anti-PF4/heparin Abs. The resulting alterations in platelet functionality were evaluated using flow cytometry.

**Figure 1:**
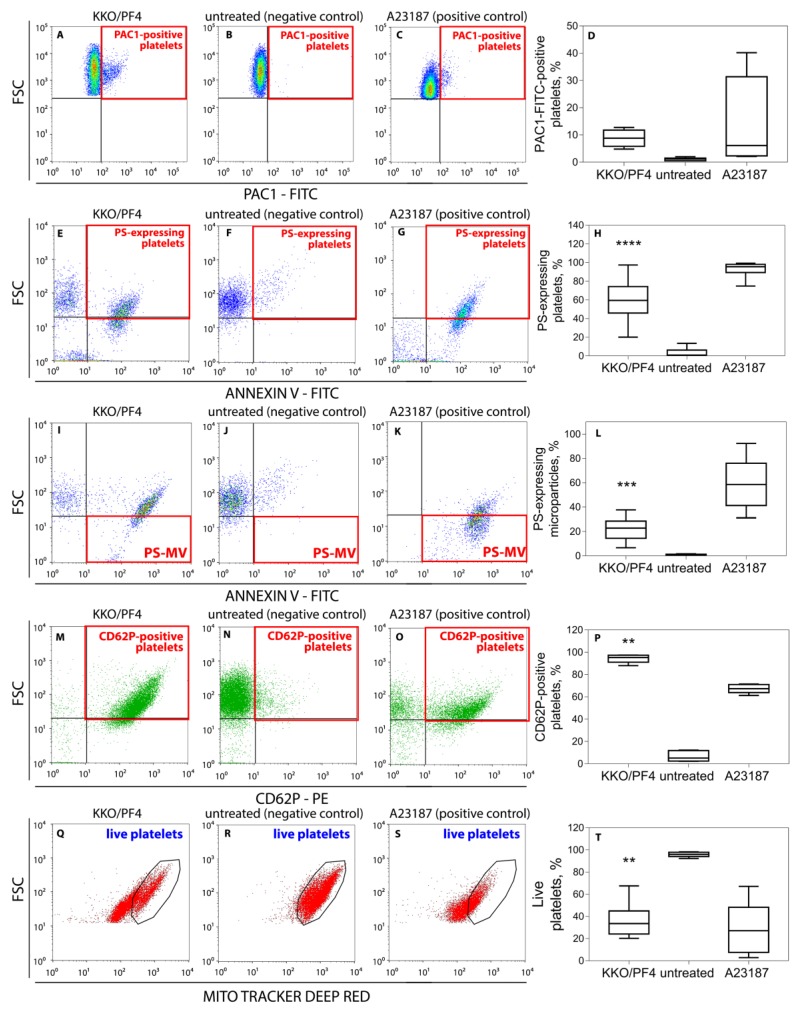
Representative dot-plots and averaged results of platelet flow cytometry under various experimental conditions. (**A**–**D**) Expression of the active integrin αIIbβ3 on the surface of platelets determined with PAC1-FITC-Abs. (**E**–**L**) Expression of phosphatidylserine on the surface of platelets **(E**–**H)** and platelet microvesicles (**I**–**L**) labeled with Annexin V-FITC. (**M**–**P**) Expression of P-selectin (CD62P) on platelets labeled with anti-CD62P-PE-Abs. (**Q**–**T**) Fractions of live platelets (gated CD41-positive particles) with normal mitochondrial membrane potential (ΔΨm) determined with a ΔΨm-sensitive dye MitoTracker Deep Red. Platelets were incubated for 60 min without (negative control) or with a mixture of KKO (pathogenic monoclonal anti-PF4/heparin Ab) (50 μg/mL KKO) and PF4 (10 μg/mL) or 10 μM calcium ionophore (positive control). Red squares indicate the quadrants and black circles (**Q**–**S**) indicate the gates with quantified signals. Each experiment was performed with platelets isolated from at least three independent donors. ** *p* < 0.01, *** *p* < 0.001, **** *p* < 0.0001 compared to a corresponding negative control (Kruskal–Wallis test with Dunn’s post-hoc test).

Quantification showed a slightly higher level of expression of the active integrin αIIbβ3 (Figure 1A–D) and a significantly higher exposure of phosphatidylserine (PS) (Figure 1E–H) under the action of pathogenic KKO and PF4 or calcium ionophore used as a positive control. Treatment of platelets with KKO/PF4 and calcium ionophore also resulted in an increased formation of PS-expressing platelet-derived microvesicles (Figure 1I–L). No significant difference was detected when comparing the expression of active αIIbβ3, PS and microvesiculation between platelets treated with PF4 mixed with RTO, or PF4, KKO or RTO alone (not shown), similar to the untreated platelets presented in Figure 1B,D (αIIbβ3); Figure 1F,H (PS); and Figure 1J,L (microvesiculation).

Platelets treated with PF4 plus KKO also displayed increased expression of P-selectin (CD62P) on the cell membrane (Figure 1M–P). The effect of the KKO/PF4 combination was similar to the calcium ionophore A23187, known as a powerful inducer of platelet activation [14], which also led to an increase in the fraction of CD62P-positive platelets. Compared to the negative control, pure PF4, KKO or RTO alone, as well as non-pathogenic Ab RTO with PF4, had no detectable effect on the P-selectin expression in platelets (not shown) and the dot-plots were similar to the untreated platelets presented on Figure 1N,P. 

Of the four parameters altered in KKO/PF4-treated platelets, as determined by flow cytometry (Figure 1A–P), the increased expression of active αIIbβ3 and P-selectin are unequivocal biochemical signs of platelet activation and degranulation, while the two others (exposure of PS and microvesiculation) may have a dual nature: they can reflect either cellular activation [15] or apoptosis [16,17,18]. Therefore, we further aimed at studying the possible death pathways of platelets under the action of the pathogenic HIT-like Abs. First, we performed flow cytometry to measure the mitochondrial transmembrane potential (ΔΨm), a general characteristic of cell viability. It was shown that after treatment for 1 h with KKO/PF4 or calcium ionophore A23187 (positive control), the fraction of platelets with reduced or lost mitochondrial transmembrane potential was significantly increased, while the fraction of platelets with normal ΔΨm was substantially reduced (Figure 1Q–T). This effect was in contrast to the negative control as well as isolated KKO, PF4, RTO, or the non-pathogenic RTO + PF4 (not shown), which maintained a high ΔΨm, similar to the untreated platelets shown in Figure 1R,T.

**Figure 2:**
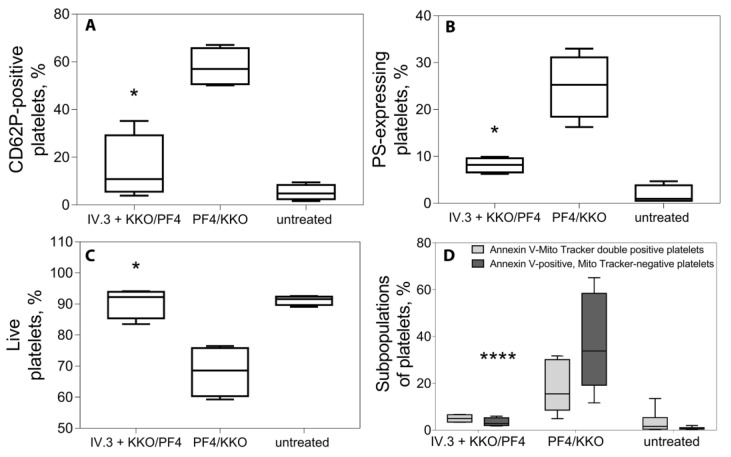
Effects of the mixture of KKO and PF4 on platelets pre-treated with a monoclonal Ab IV.3 against the FcγRII-receptor. Platelets were incubated for 60 min at 37 °C under the following conditions: untreated platelets (negative control), treated with KKO/PF4 (positive control) and treated with KKO/PF4 after pre-treatment for 10 min at 37 °C with a monoclonal Ab IV.3 against the FcγRII-receptor. Final concentrations: 10 µg/mL PF4, 50 µg/mL KKO, 50 µg/mL IV.3. (**A**) Fraction of platelets expressing P-selectin (CD62P-positive). (**B**) Fraction of platelets expressing phosphatidylserine (Annexin V-positive). (**C**) Fraction of live platelets with normal mitochondrial membrane potential determined with a ΔΨm-sensitive (mitochondrial membrane potential) dye MitoTrackerDeepRed. (**D**) Subpopulations of platelets double-stained with a ΔΨm-sensitive dye MitoTrackerDeepRed and FITC-Annexin V normalized by the total number of gated platelets taken as 100%. Platelets were segregated into 2 subpopulations: MitoTracker-negative/Annexin V-positive or “dead platelets” (dark boxes) and MitoTracker-positive/Annexin V-positive or “live activated platelets” (light boxes). Each experiment was performed with platelets isolated from three independent donors. * *p* < 0.05, **** *p* < 0.0001 for the corresponding data without and with the addition of Ab IV.3 (A-C, Kruskal–Wallis test; D, 2-way ANOVA test with Dunn’s post-hoc test).

To confirm that the described effects of the KKO/PF4 were mediated by the platelet FcγRIIA receptor, the measurements were performed in the presence of an FcγRIIA-blocking monoclonal Ab (mAb IV.3). Importantly, suppression of FcγRIIA reduced the level of P-selectin and PS expression down to the baseline, indistinguishable from the background control values in unstimulated platelets, indicating that platelet activation with KKO/PF4 was mediated by the FcγRIIA receptor (Figure 2A,B). This was consistent with preservation of ΔΨm (Figure 2C) and a decrease in the subpopulation of dying platelets after blockage of the FcγRIIA receptor (Figure 2D). 

**Figure 3:**
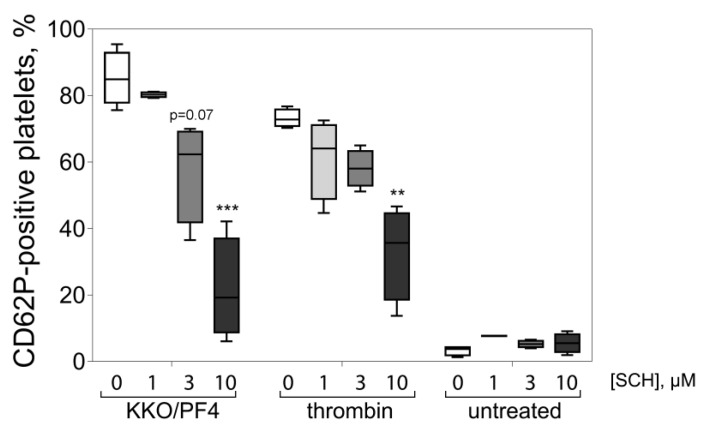
Inhibitory effect of SCH 79797, a selective antagonist of PAR1 receptors, on platelet activation induced by the combination of KKO and PF4. Platelets were pre-incubated for 30 min at 37 °C with SCH 79797 at 0, 1, 3, and 10 μM followed by treatment for 60 min at 37 °C with KKO/PF4 (10 and 50 µg/mL, respectively), 1 U/mL thrombin (positive control), and without any treatment (untreated, negative control). Platelet activation was assessed by the expression of P-selectin, determined using flow cytometry with fluorescently labeled anti-CD62P-Abs. Each experiment was performed with platelets isolated from three independent donors. ** *p* < 0.01, *** *p* < 0.001 compared to a corresponding control without addition of SCH 79797 (2-way ANOVA test with Dunn’s post-hoc test).

Interestingly, the increased expression of P-selectin induced by KKO/PF4 was prevented by the direct inhibition of protease-activated receptors 1 (PAR1) receptors in a dose-dependent manner (Figure 3), indicating that some of the activating effects of KKO/PF4 on platelets are secondary due to thrombin generation on or near the surface of platelets primarily activated by KKO/PF4.

Thus, the pathogenic Abs combined with platelet factor 4 caused not only pronounced activation of platelets, but also induced the signs of mitochondrial dysfunction and cell death. These effects were mediated by the direct interaction of the pathogenic KKO mixed with PF4 with the FcγRIIA receptors as well as via secondary activation of PAR1 receptors by thrombin formed on the activated platelets. Next, we explored if KKO/PF4-treated platelets could undergo apoptotic death pathway.

### 2.2. Apoptotic Markers in Platelets induced by KKO/PF4

Although platelets have no nucleus, they can undergo apoptosis via a mitochondrial pathway that involves up- or down-regulation of mitochondrial Bcl-2 family proteins that have anti- or pro-apoptotic activity [19]. Since flow cytometry data suggest the possibility of apoptosis induced by the PF4-containing complexes, we used Western blot analysis to test this assumption by quantifying apoptotic molecular markers. We quantified the expression of Bax and Bcl-X_L_, the components of the *Bcl-2* gene family that are known to be expressed in platelets [20]. Bcl-X_L_ is an anti-apoptotic protein that modulates apoptosis by controlling mitochondrial membrane permeability and regulating the release of cytochrome c [21]. The pro-apoptotic Bax protein is involved in the regulation of apoptosis by the formation of large oligomers in the outer mitochondrial membrane, which promote cell apoptosis. Bax contributes to the release of cytochrome *c* into the cytosol, which in turn triggers the activation of caspases [22]. Accordingly, the activation/cleavage of pro-caspase-3 is another characteristic sign of platelet apoptosis and Western blot analysis with an anti-caspase-3 Ab can show whether 32-kDa pro-caspase-3 is cleaved into a 17-kDa fragment representing active caspase-3. 

**Figure 4:**
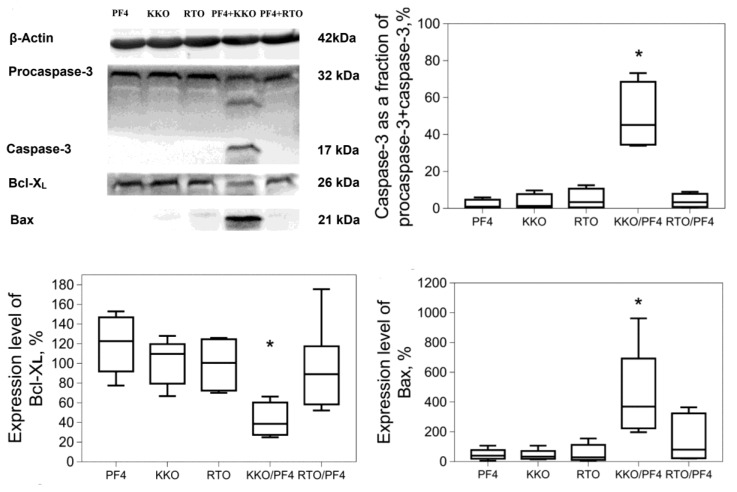
Western blot analysis of molecular markers of apoptosis [(pro)caspase-3, Bcl-X_L_, Bax] in platelet lysates obtained under various experimental conditions. Platelets were incubated for 60 min with 10 μg/mL PF4, 50 μg/mL KKO (pathogenic monoclonal anti-PF4/heparin Ab), 50 μg/mL RTO (non-pathogenic monoclonal anti-PF4/heparin Ab), as well as KKO + PF4 and RTO + PF4 combinations. The figure shows representative Western blots and quantitative analysis of the molecular markers of apoptosis based on four experiments with platelets isolated from independent donors. The numbers that characterize the size and intensity of each band were normalized by the corresponding negative control (untreated platelets) taken as 100%. * p < 0.05 compared to RTO/PF4 (Kruskal–Wallis test with Dunn’s post-hoc test).

Western blot analysis showed that a 17-kDa fragment representing activated caspase-3 appeared in the platelets treated with KKO/PF4 and comprised 50% ± 18% (M ± SEM) of the total pro-caspase 3 + caspase 3 levels (Figure 4). Such a band was not formed under any other experimental conditions except for the calcium ionophore A23187, which induced almost complete conversion of the pro-caspase 3 to the active cleaved caspase 3 (not shown). The expression of the anti-apoptotic protein Bcl-X_L_ was significantly reduced, while the level of the pro-apoptotic protein Bax was substantially increased in the platelets treated with the pathogenic KKO + PF4 complexes as opposed to control untreated platelets (Figure 4). 

Taken together, the results show that the KKO/PF4 mixture induces platelet apoptosis that is characterized by mitochondrial dysfunction combined with up-regulation of the pro-apoptotic protein Bax, down-regulation of the anti-apoptotic protein Bcl-X_L_, and moderate activation of procaspase-3. In view of these data, the high level of PS expression on the platelet outer membrane, as well as the formation of microvesicles, may be considered not only signs of platelet activation, but also signatures of platelet death [23,24], as in nucleated cells [25]. Therefore, the apoptotic death pathway can be a pathogenic mechanism of thrombocytopenia in HIT. 

### 2.3. KKO/PF4-induced Calpain Activation

In addition to the caspase 3 activation, we analyzed the activity of calpains, a family of calcium-dependent cysteine proteases that have been shown to play important roles in platelet functions, such as aggregation, adhesion, spreading, and platelet-driven contraction of blood clots [26]. In addition, high calpain activity has been shown to be associated with non-apoptotic platelet death induced by thrombin [9]. 

**Figure 5:**
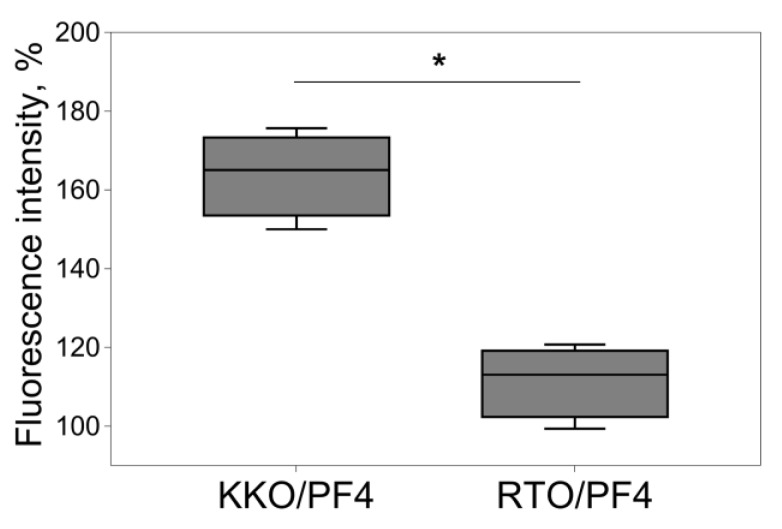
Fluorimetric analysis of calpain activity in platelets that were incubated for 180 min with a mixture of 10 μg/mL PF4 + 50 μg/mL KKO or RTO. The numbers that characterize the calpain activity were normalized by the corresponding negative control (untreated platelets) taken as 100%. Experiments were performed with platelets isolated from seven independent donors. * *p* < 0.05 (Mann–Whitney U test).

The fluorimetry of platelets in the presence of a fluorogenic calpain substrate revealed that the treatment of platelets with KKO/PF4 induced the activation of calpain with a maximum activity at 180 min compared to the RTO/PF4-treated platelets (Figure 5). The results indicate that HIT-pathogenic Abs in the presence of PF4 induce a non-apoptotic death pathway associated with calpain activation.

In addition to the previously published paper [27], the present manuscript contains new evidence for the existence of high calpain activity associated with the apoptotic death pathway of platelets treated with KKO/PF4. Therefore, we conclude that HIT-like immune complexes cause platelet death through complex apoptotic and non-apoptotic pathways.

## 3. Materials and Methods 

### 3.1. Platelet Isolation and Incubation with Immune Complexes Containing PF4

Platelets were freshly isolated from the blood of 55 healthy donors not taking medications that affect platelet function for at least two weeks before the blood withdrawal. Informed consent from all the blood donors was obtained. All procedures were performed in accordance with the guidelines approved by the Ethical Committee of Kazan State Medical Academy (Kazan, Russian Federation). Venous blood was collected into 3.2% trisodium citrate tubes (9:1) and immediately centrifuged at room temperature at 200g for 10 min to obtain platelet-rich plasma (PRP). Platelets from PRP were isolated by gel filtration at room temperature on Sepharose 2B equilibrated with Tyrode’s buffer (4 mM HEPES, 135 mM NaCl, 2.7 mM KCl, 2.4 mM MgCl_2_, 5.6 mM D-glucose, 3.3 mM NaH_2_PO_4_, 0.35 mg/mL bovine serum albumin, pH 7.4). The fraction of untreated platelets activated during isolation did not exceed 10%, as determined by expression of the activated integrin αβIIb3, PS or P-selectin (Figure 1B,F,N).

Platelets in Tyrode’s buffer with the addition of 2 mM CaCl_2_ were incubated at 37 °C for 15, 60 and 180 min with PF4 (10 μg/mL) and HIT-like pathogenic monoclonal Ab KKO [10] at 50 μg/mL or anti-PF4 non-pathogenic monoclonal Ab RTO (50 μg/mL) [11]. In a series of inhibitory experiments; platelets were pre-treated with 50 µg/mL of a monoclonal Ab against the Fcγ receptor IIA (anti-FcγRIIA; clone IV.3) [28] or with 1, 3, and 10 µM of a selective antagonist of PAR1 receptors (SCH 79797, Sigma-Aldrich, St. Louis, MO, USA) that were added to platelets for 15 min (for anti-FcγRIIA) and 60 min (for SCH 79797) [29] prior to the incubation with the mixture of KKO and PF4. Untreated platelets and platelets incubated with either PF4, KKO or RTO alone were used as negative controls and platelets incubated with 10 µM calcium ionophore A23187 were used as a positive control. KKO, RTO and recombinant PF4 were obtained as previously described [28].

### 3.2. Flow Cytometry

**Figure 6:**
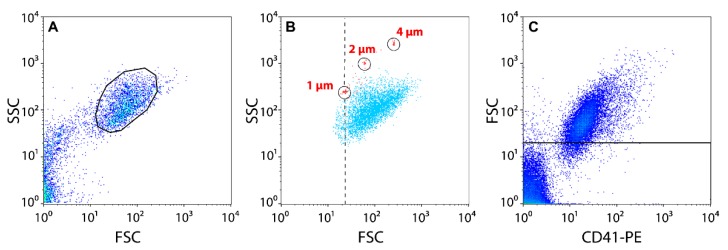
Flow cytometry of isolated platelets. (**A**) Platelets were gated (black circle) based on the size and granularity using Forward Light Scatter (FSC) and Side Scatter (SSC) channels. (**B**) The lower size limit of the platelet gate in FSC/SSC dot-plots (dashed line) was established using 1-, 2-, and 4-µm latex beads. (**C**) In addition to the FSC/SSC scatter, platelets were gated (above the line) using anti-CD41-PE-labeled Abs.

Platelets were gated in a flow cytometer by their forward scatter/fide scatter (FSC/SCC) characteristics after size-based calibration with 1-, 2-, and 4-μm polystyrene beads (Figure 6A,B). About 97% of events in the gate were CD41-positive (Figure 6C). Platelet-derived microvesicles were identified as the events that reflected particles <1 µm in size as positive for the platelet-specific marker CD41. For each sample analyzed, 30,000 events were collected using a FacsCalibur flow cytometer (BD Biosciences, USA) equipped with an argon laser (λ = 488 nm) and a diode red laser (λ = 635 nm). The data were analyzed using CellQuest Pro (BD Biosciences) and FlowJo software. Specific markers on platelets were identified by labeling with PE-conjugated monoclonal Abs to platelet-specific antigen CD41, FITC-conjugated (fluorescein isothiocyanate) PAC-1 Abs against activated αIIbβ3 (BD Biosciences, Franklin Lakes, NJ, USA), and Annexin V conjugated with FITC to assess the expression of PS on the platelet membrane. Evaluation of the expression of P-selectin (CD62P) on the platelet surface was performed by the addition of murine anti-CD62P Abs labeled with a fluorescent dye phycoerythrin (PE). Changes in the mitochondrial membrane potential (ΔΨm) were assessed after adding the ΔΨm-sensitive lipophilic cationic carbocyanine-based fluorochrome (MitoTracker Deep Red FM). The fraction of platelets fluorescently labeled by a corresponding marker was determined from the total number of platelets in the gate.

### 3.3. Western Blot Analysis

Platelets incubated under various experimental conditions were supplemented with a lysis RIPA buffer (pH 7.4) containing a proteinase and phosphatase inhibitor cocktail, and the mixture was incubated at 4 °C overnight with continuous shaking. Cell lysates were subjected to gradient 12%–20% SDS-PAGE (sodium dodecyl sulfate–polyacrylamide gel electrophoresis) under denaturing conditions. After protein transfer, the nitrocellulose membranes (BioRad, Hercules, CA, USA) were incubated overnight with Abs against human (pro)caspase 3 (Millipore Sigma, USA, cat. # MAB4703, clone 4-1-18), Bax (BioLegend, San Diego, CA, USA, cat. # 633602) and Bcl-XL (Abcam, Cambridge, UK, cat. # ab201335). To detect the primary Abs, blots were incubated with the secondary goat horseradish peroxidase-conjugated anti-mouse IgG Abs (Invitrogen, Waltham, MA, USA).

### 3.4. Assay of Calpain Activity

Isolated platelets were pre-incubated with 10 µM Calpain Substrate IV (Calbiochem, San Diego, CA, USA) followed by incubation with the combinations of KKO + PF4 and RTO + PF4, as well as isolated PF4, KKO or RTO, at the concentrations shown above. The fluorescence intensity of EDANS (5-((2-Aminoethyl)amino)naphthalene-1-sulfonic acid) released from the calpain substrate was measured at 380 ± 20 nm excitation and 465 ± 30 nm emission wavelengths on a multimode plate reader Infinite F Plex (Tecan, Männedorf, Switzerland). Ca^2+^-ionophore A23187 (10 μM) used as a positive control caused a strong 8-9-fold activation of calpain in platelets after 3-h incubation (not shown).

### 3.5. Statistical Analysis

Statistical analyses were performed using a Prism 7.0 software package (GraphPad Software, San Diego, CA, USA). Statistical differences were estimated using the nonparametric Kruskal-Wallis test with Dunn’s post-hoc test, 2-way ANOVA with Dunn’s post-hoc test after checking the data for normality using Shapiro–Wilk and KS normality criteria, and Mann–Whitney U test for a pairwise comparison with a 95% level of confidence. The results are presented as a median and intervals between the 25th and 75th, as well as between the 5th and 95^th^, percentiles.

## 4. Conclusions

Platelet activation induced by pathogenic complexes proceeds directly through the FcγRIIA receptors or indirectly via PAR1 receptors. The effect of KKO/PF4 is accompanied by platelet dysfunction and death via complex apoptotic and calpain-dependent, non-apoptotic pathways. These platelet death pathways may comprise pathogenic mechanisms leading to the persistent low platelet count in HIT. 

## Abbreviations

HIT PF4	Heparin-induced thrombocytopenia Platelet factor 4
PS ADP KKO RTO ΔΨm CD62P mAb IV.3 PAC1	Phosphatidylserine Adenosine diphosphate Pathogenic monoclonal anti-PF4/heparin antibody Non-pathogenic monoclonal anti-PF4/heparin antibody Mitochondrial membrane potential P-selectin FcγRIIA-blocking antibody, clone IV.3 Monoclonal antibody reacting with the active integrin αIIbβ3
PRP FITC FSC/SSC PE SDS-PAGE EDANS PAR1	Platelet-rich plasma Fluorescein isothiocyanate Forward scatter/Side scatter Phycoerythrin Sodium dodecyl sulfate–polyacrylamide gel electrophoresis 5-((2-Aminoethyl)amino)naphthalene-1-sulfonic acid Protease-activated receptor 1
